# High-Throughput Cloning and Characterization of Emerging Adenovirus Types 70, 73, 74, and 75

**DOI:** 10.3390/ijms21176370

**Published:** 2020-09-02

**Authors:** Wenli Zhang, Kemal Mese, Sebastian Schellhorn, Nora Bahlmann, Nicolas Mach, Oskar Bunz, Akshay Dhingra, Elias Hage, Marie-Edith Lafon, Harald Wodrich, Albert Heim, Anja Ehrhardt

**Affiliations:** 1 Virology and Microbiology, Center for Biomedical Education and Research (ZBAF), Department of Human Medicine, Faculty of Health, Witten/Herdecke University, 58453 Witten, Germany; wenli.zhang@uni-wh.de (W.Z.); Kemal.Mese@uni-wh.de (K.M.); Sebastian.Schellhorn@uni-wh.de (S.S.); norabahlmann@googlemail.com (N.B.); Nicolas.Mach@uni-wh.de (N.M.); Oskar.Bunz@uni-wh.de (O.B.); 2 Institut für Virologie, Adenovirus Konsiliarlabor, Medizinische Hochschule, 30625 Hannover, Germany; Dhingra.Akshay@mh-hannover.de (A.D.); hage.elias@hotmail.com (E.H.); heim.albert@mh-hannover.de (A.H.); 3 Microbiologie Fondamentale et Pathogénicité, Université de Bordeaux, 33076 Bordeaux, France; marie-edith.lafon@u-bordeaux.fr (M.-E.L.); harald.wodrich@u-bordeaux.fr (H.W.)

**Keywords:** emerging adenoviruses, genetic access, linear-linear homologous recombination, high-throughput cloning, receptor usage

## Abstract

Recently an increasing number of new adenovirus types associated with type-dependent pathogenicity have been identified. However, identification of these clinical isolates represents the very first step to characterize novel pathogens. For deeper analyses, these adenoviruses need to be further characterized in basic virology experiments or they could be applied in translational research. To achieve this goal, it is essential to get genetic access and to enable genetic modification of these novel adenovirus genomes (deletion, insertion, and mutation). Here we demonstrate a high-throughput approach to get genetic access to new adenoviruses via homologous recombination. We first defined the cloning conditions regarding homology arm-length and input adenoviral genome amounts. Then we cloned four naturally occurring adenoviruses (Ad70, Ad73, Ad74, and Ad75) into easy-to-manipulate plasmids and genetically modified them by reporter gene insertion. Three recombinant adenoviruses (Ad70, Ad73, and Ad74) containing a reporter cassette were successfully reconstituted. These novel reporter-labeled adenoviruses were further characterized using the inserted luciferase reporter with respect to receptor usage, presence of anti-adenovirus antibodies, and tropism in vitro. The identified receptor usage, the relatively low prevalence of anti-adenovirus antibodies, and the various cancer cell line transduction pattern are important features of these new pathogens providing essential information for their therapeutic application.

## 1. Introduction

Adenoviruses are medium-sized (70–100 nm in diameter), non-enveloped icosahedral viruses composed of double-stranded linear DNA genome with an average length of 26–45 kb [1]. In humans more than 100 adenovirus types have been identified and classified into seven species (A to G) based on hemagglutination properties, oncogenicity in rodents, DNA homology, and genomic organization [2,3]. Human adenoviruses (HAdVs) cause significant numbers of respiratory, ocular, and gastrointestinal diseases and incidences of severe diseases caused by adenoviruses most occur in immunocompromised individuals or toddlers [4,5,6]. Recent outbreaks of adenovirus infections are at least partly due to newly evolving adenovirus types from constant HAdVs molecular evolution [2,7,8]. To identify these novel clinical isolates is the very first step to study a new pathogen. However, for further studies, it is essential to get genetic access and to enable genetic modification of these novel adenovirus genomes.

Since the first discovery in the 1950s [9], adenoviruses have long been used as model systems to study cellular and viral processes as well as gene transfer tool for gene therapy, as vaccine vector or as oncolytic virus [3,10,11]. In the moment, adenovirus-based vaccines are the most promising solution to stop COVID-19. The two phase ½ and phase 2 trials from the Oxford COVID Vaccine Trial Group and CanSino Biologics have demonstrated potent humoral and cellular immune responses, and both are going to be evaluated in phase 3 trial [12,13]. However, the broad application of commonly used adenoviral vectors based on adenovirus types 2 and 5 (Ad2 and Ad5) is highly limited by restricted receptor usage and pre-existing immunity [14,15]. Therefore, there is a high interest in the research community to work with other serotypes such as Ad3, 26, and 35 [16,17]. However, the further exploration of the more than 100 human adenovirus types was hindered for a long time by lack of convenient genetic modification methods. To convert adenoviruses to vectors, several strategies have been devised, such as cosmid-based methods, traditional cut- and-paste based molecular cloning, homologous recombination in bacteria or in eukaryotic cells (summarized in review [18]), and most recently described Gibson gene assembly technique [19,20]. Those methods were either time consuming or complicated.

To get genetic access to novel emerging adenoviruses more confidently, we previously developed RecET-mediated linear-linear homologous recombination (LLHR) to incorporate the adenoviral genomes into easy-to-manipulate plasmids [21,22]. This method is solid and enabled efficient cloning of every tested adenovirus genome isolated from purified virus. However, when we tried to move one step further, to clone adenovirus genomes directly from virus infected cells, it was not consistently successful. Therefore, in the current study, we first optimized and defined the adenovirus genome cloning conditions. Based on this knowledge, four recently isolated species D adenoviruses Ad70 [23], Ad73, Ad74, and Ad75 [24] were cloned and further modified by reporter gene insertion. With these reporter-inserted novel adenoviruses, we then performed screening assays to characterize their receptor usage, the prevalence of anti-adenoviral antibodies and the transduction efficiencies of various cancer cell lines.

## 2. Results

The main purpose of this study was to optimize the adenovirus genome cloning condition to facilitate the cloning from infectious material. The process was then exemplified by cloning and modification of four newly isolated HAdVs (Ad70, Ad73, Ad74, and Ad75). 

### 2.1. Role of Homology Arm-Length in the Efficiency of Adenoviral Genome Direct Cloning

**Figure 1:**
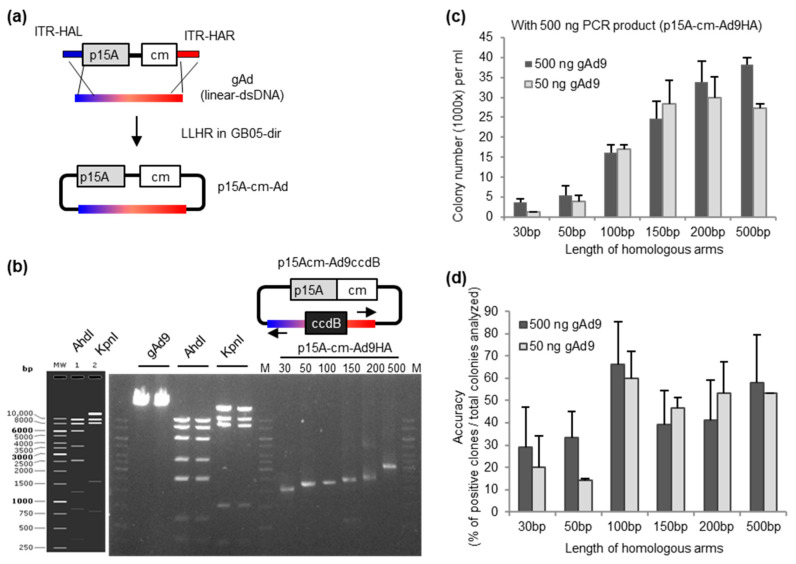
Homology arm-length dependence of adenoviral genome (gAd) cloning efficiency. (**a**) Schematic diagram illustrating the strategy for direct cloning of gAd isolated from cesium chloride (CsCl) purified adenovirus. The linear p15A-cm vector and adenoviral genome contain identical sequences at both ends (ITR-HAL and ITR-HAR) as homology arms (HA) for direct cloning. The gAds were cloned into the linear p15A-cm vector by linear-linear homologous recombination (LLHR) using the *E. coli* strain GB05-dir. (**b**) A polymerase chain reaction (PCR) product containing p15A and the chloramphenicol resistance gene (cm) flanked by adHAs was used for direct cloning. The plasmid p15Acm-Ad9ccdB was used as template to perform the PCR, generating a cloning vector with various HA length (30-, 50-, 100-, 150-, 200-, or 500-bp) (Figure S1). The gAd9 was digested either with AhdI or KpnI as verification. On the left panel is the simulated gel picture of the digest, while the actual digest is shown on the gel in the right panel. In the first two lanes are the isolated adenovirus type 9 genomes (gAd9). Then the digested gAd9 and PCR products are depicted. M, 1 Kb DNA marker (VWR, Langenfeld, Germany). Five hundred nanograms of the PCR-generated linear vector flanked by 30-, 50-, 100-, 150-, 200-, or 500-bp homology arms was co-transformed with either 50 ng or 500 ng of gAd9 into the *E. coli* strain GB05-dir. The cloning efficiency is expressed via colony number counting (**c**), while accuracy via percentage of positive colonies detected by colony PCR (Figure S2) (**d**).

The efficiency of adenoviral genome direct cloning can be influenced by the length of homology arm (HA). We evaluated a variety of HA-lengths using a pilot experiment based on direct cloning of human adenovirus 9 genome (gAd9) (Figure 1). A series of linear cloning vectors with wide spectrum HA-lengths (30-, 50-, 100-, 150-, 200-, or 500-bp) were generated by polymerase chain reaction (PCR). To avoid cloning background contamination from the PCR, a previously generated plasmid p15Acm-Ad9ccdB [22] was used as template (Figure S1). Five hundred nanograms or 50 ng of gAd9 were co-electroporated with 500 ng individual cloning vectors varying regarding the HA length. The cloning efficiency was compared using the total colony number on antibiotic selection. The gAd cloning was successful with HA lengths as short as a 30 bp with both high (500 ng) and low (50 ng) amounts of gAd9. The cloning efficiency could be enhanced significantly as the HA length increased from 30 to 150 bp. The extension of HA length from 150 to 200 and 500 bp resulted in relative steady level of colony numbers.

The cloning product quality determined by the fidelity of homologous recombination was also carefully evaluated. We first established a colony-PCR to detect gAd9 and the PCR was validated by including primer pairs amplifying the cloning vector backbone. The HA-length of 30 and 50 bp led to 20% to 30% positive clones, while the HA-length above 100 bp gave in average around 50% of positive clones. It is of note, the cloning efficiency using HA100 was the highest among all tested HA lengths, indicating that HA100 represents the optimal setup. After assessing the accuracy via colony-PCR, we chose a few representative positive and negative clones for culture and plasmid isolation for restriction analysis (Figure S2). From 23 colony-PCR detected positive clones, the DNA digest pattern of one clone (number 7 of the HA150 bp) was shown to not be correct, probably due to intra-plasmid recombination resulting in incomplete gAd9. Furthermore, the cloning junction was confirmed by sequencing. 

### 2.2. Sufficient Amount of Adenovirus Genome Is Required for Successful Cloning

**Figure 2:**
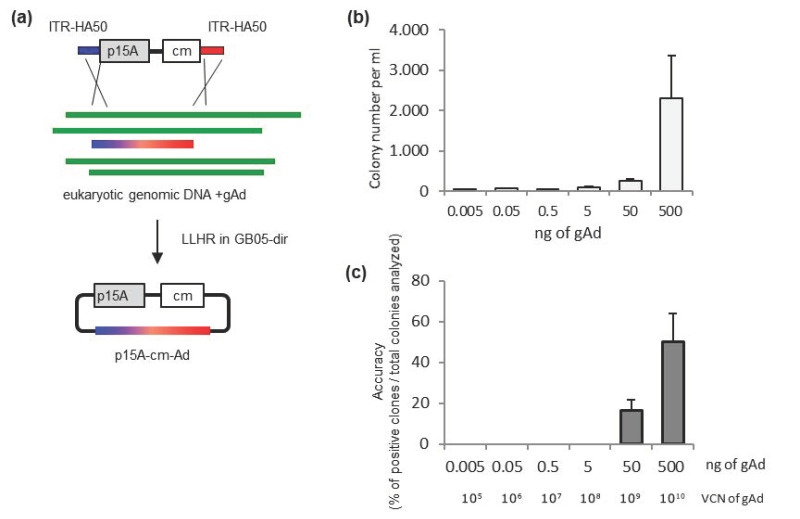
Influence of adenoviral genome (gAd) amount in cell/virus mixture on cloning efficiency. (**a**) Schematic diagram illustrating the setting for direct cloning of gAd from a DNA mixture with eukaryotic genomic DNA from A549 cells. The linear p15A-cm vector and adenoviral genome have identical sequences at both ends (ITR-HA) as homology arms for direct cloning. Five hundred nanograms of the linear vector flanked by 50-bp homology arms (p15A-cmAd9HA) was co-transformed with either 0.005, 0.05, 0.5, 5, 50, or 500 ng of gAd9 diluted in either 500, 499.9, 499.5, 495, 450, or 0.005 ng eukaryotic genomic DNA. The cloning efficiency is expressed by obtained colony numbers (**b**), while accuracy via percentage of positive colonies detected by colony PCR (Figure S2) (**c**). VCN, virus genome copy number, calculated in an online DNA copies calculator from URI Genomics and Sequencing Center (http://cels.uri.edu/gsc/cndna.html).

A second critical factor limiting gAd cloning is the molecular input DNAs. For the cloning vector generated by PCR, the amount can be easily adjusted. As to the other essential component (the gAd DNA), however, its amount can be limited in case of a clinical sample. Therefore, we set a second experiment to evaluate the influence of the gAd amount in the cloning process (Figure 2). To simulate the clinical sample situation, the gAd9 was pre-diluted and mixed with eukaryotic genomic DNA isolated from A549 cells. After co-electroporation of the genomic DNA mixture and cloning vector into competent GB05-dir bacteria, the cloning efficiency and accuracy were evaluated. The dilution from 500 ng to 0.005 ng gAd9 are equivalent to virus genome copy numbers from 10^10^ to 10^5^. The reduction of gAd9 from 500 ng to 50 ng caused the colony number to drop 10-fold, associated with a two third loss of accuracy. While minimal amounts of colony numbers in samples which received the further diluted gAd9 (from 5 to 0.005 ng) were counted, no single positive colony in these settings was detected. 

### 2.3. High-Throughput Cloning of Emerging Adenoviruses Types 70, 73, 74, and 75

**Figure 3:**
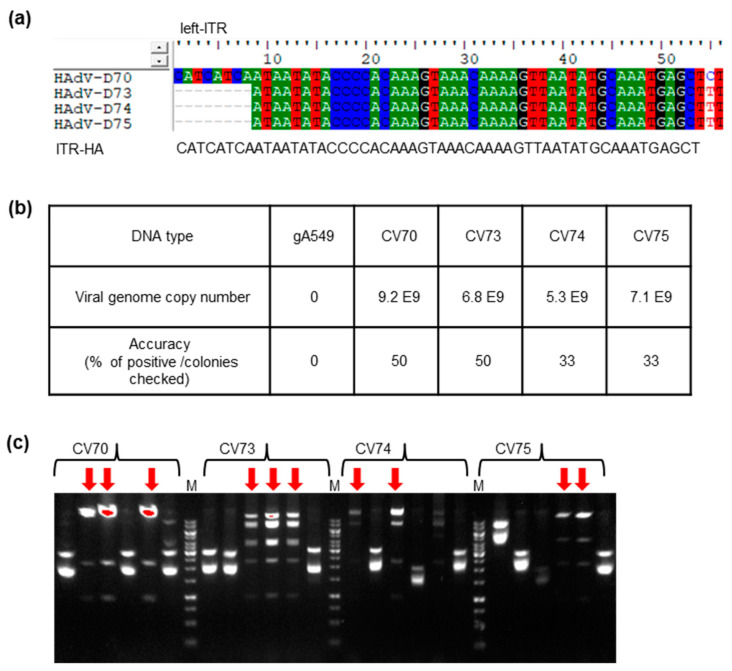
High-throughput cloning of new species D human adenoviruses (HAdV-D). (**a**) Identification of conserved sequences in the ends of inverted terminal repeats (ITR). Genome sequences of four new HAdV-Ds (Ad70, Ad73, Ad74, and Ad75) were analyzed by ClustalW Multiple alignment in BioEdit Sequence Alignment Editor (https://bioedit.software.informer.com/7.2/). The conserved ITR end sequence was used as homologous arm (ITR-HA) for cloning vector construction. (**b**) Viral genome copy number-dependent cloning efficiency. Linear-linear homologous recombination (LLHR) was performed with 500 ng of cloning vector p15A-cm-DHA50 and genomic DNA: gA549, genomic DNA isolated from A549 cells; CV70, CV73, CV74, and CV75, cell and virus mixture, genomic DNA isolated from individual adenovirus (Ad70, Ad73, Ad74, and Ad75) infected A549 cells. (**c**). HindIII restriction analysis of the cloned adenoviral genome in p15A. Correct clones are indicated with red arrows. M, 1 Kb DNA marker.

The species D adenoviruses, which represent the largest species of HAdVs consists of a high amount of homologous DNA sequences. As shown in Figure 3, the studied adenovirus types in the present study (70, 73, 74, and 75) have identical inverted terminal repeat (ITR) at the 5′ and 3′ ends of the viral genome. This facilitates to perform high-throughput cloning using one cloning vector. Here a linear cloning vector flanked with 50 bp HA was PCR generated. Respective clinical isolates of adenoviruses were cultured in A549 cells. Two days after infection, virus infected cells were collected for total genomic DNA isolation. The gAd cloning was performed as displayed in the scheme in Figure 2. The gAd copy number in the samples was quantified via real-time PCR, which later allows correlation to the cloning accuracy. Identification of correct clones by restriction analysis is presented. Of the cell/virus DNA mixture (CV70, CV73, CV74, and CV75) as gAd input, various analyzed clones were positive. From these identified positive clones, wild type adenoviruses can be rescued, and further genetic modification can be conducted. 

### 2.4. High-Throughput Reporter Insertion

**Figure 4:**
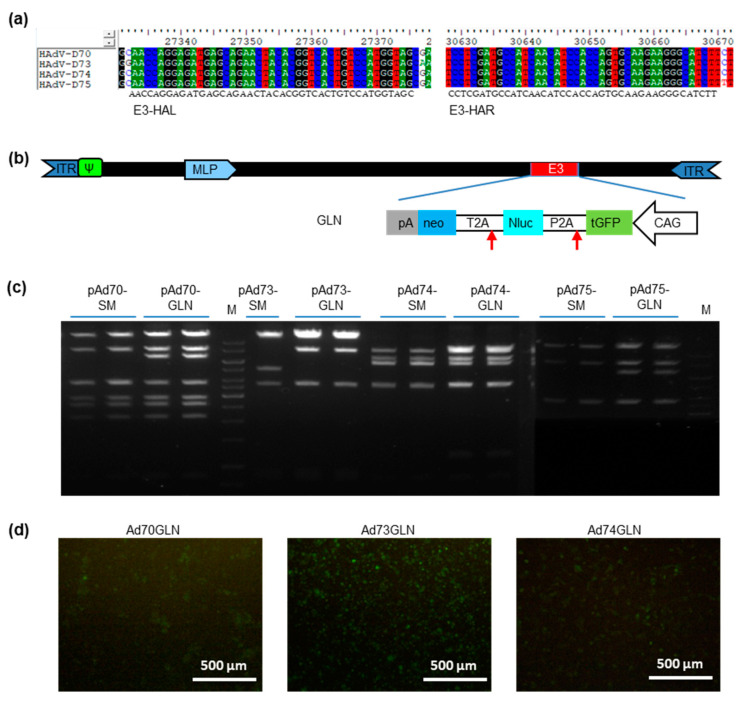
High-throughput reporter insertion in new species D human adenovirus (HAdV-D). (**a**) Identification of conserved sequences in the adenovirus early gene 3 (E3). Genome sequences of four new HAdV-Ds (Ad70, Ad73, Ad74, and Ad75) were analyzed by ClustalW Multiple alignment in BioEdit Sequence Alignment Editor, the conserved sequences on the left and right end of E3 was used as homologous arm (E3-HAL and E3-HAR) for reporter insertion. (**b**) Diagram shows the GLN reporter cassette insertion into the E3 genomic region. GLN reporter cassette expresses turbo Green fluorescent protein (GFP), nanoLuciferase (Nluc), and neomycin resistance (neo) under the control of the CAG promoter (CMV enhancer, chicken beta-Actin promoter, and rabbit beta-Globin splice acceptor site). (**c**) AhdI restriction check of the reporter insertion. SM, selection marker used for GLN insertion. M, 1 Kb DNA marker. (**d**) GFP images showing rescue of reporter-labeled adenoviruses in HEK 293 cells. Scale bar 500 µm.

To empower further studies with these new species-D HAdVs, a reporter cassette was inserted into the viral genome. Due to the high homology in species D, identical sequences in early gene 3 (E3) region was used as homologous arm for reporter gene insertion (Figure 4). A dual-reporter cassette (GLN) including green fluorescent protein (GFP) and luciferase was inserted into E3 in reverse direction to the adenovirus major late prompter (MLP). This high-throughput reporter gene-insertion cloning was successful for Ad70, Ad73, Ad74, and Ad75. Recombinant virus was rescued in HEK 293 cells for Ad70GLN, Ad73GLN, and Ad74GLN. Ad75GLN failed to be rescued. After rescue, these recombinant adenoviruses were serial amplified to large scale and purified via CsCl gradient. In the following studies, the purified viruses were applied.

### 2.5. Screening of Adenovirus Tropisms

**Figure 5:**
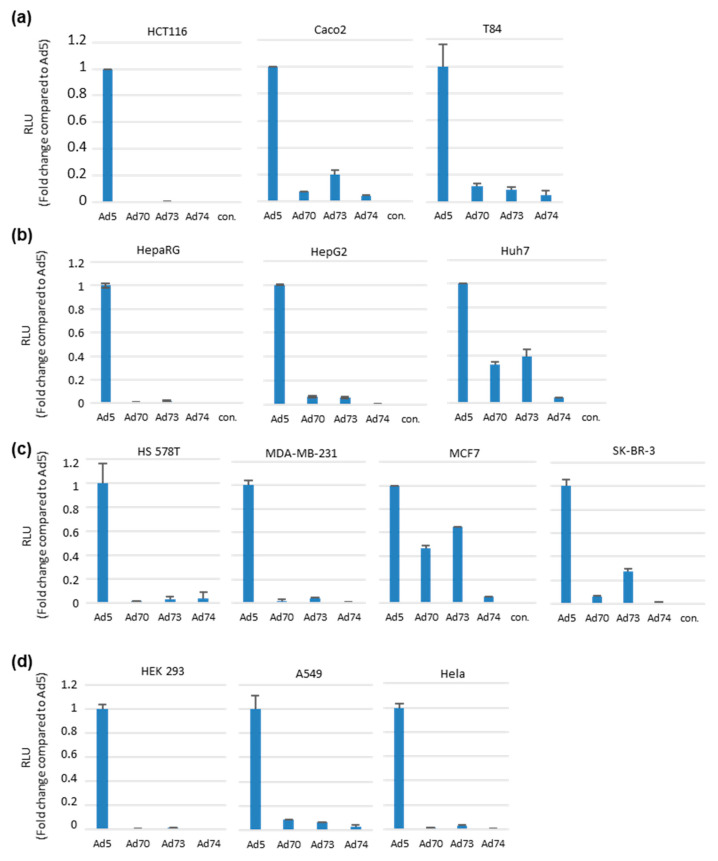
Screening of the reporter-tagged new species D human adenoviruses (HAdV-D) in human tumor cells. Transgene expression efficiency of different adenovirus types (Ad type number) was tested in a panel of disease-specific cell lines. Shown are (**a**) colon carcinoma derived cell lines, (**b**) hepatocellular carcinoma derived cell lines, (**c**) breast cancer derived cell lines, and (**d**) typical adenovirus propagation cell lines. Cells were infected at 100 viral particles per cell, luciferase expression was measured 24 h post infection by addition of furimazine substrate and expressed as relative light units (RLU). Levels were compared to the commonly used adenovirus type 5 (Ad5) and indicated as fold change. In all cell lines, error bars represent mean ± SD (*n* = 3).

To analyze these new species D adenoviruses with respect to their tropism in vitro, we applied these reporter-labeled adenoviral vectors to cell lines deriving from diverse organs (Figure 5). Transduction efficiencies evaluated by luciferase reporter assays were standardized by comparison with the commonly used Ad5 vector. The Ad5 vector containing the same reporter cassette in its E3 region was previously generated following the same cloning procedure [22]. Considering all three adenoviruses Ad70, Ad73, and Ad74 were isolated from diarrheal feces of immunocompromised patients, it was interesting to check their transduction efficiencies in gastrointestinal-derived cells. Therefore, these new viruses were applied to colon carcinoma originated cell lines HCT116, Caco2, and T84. In HCT116 cells, the three new viruses resulted in minimal transduction rates compared to Ad5, while in the Caco2 and T84 cell lines we observed around 10–20% of transduction efficiencies if directly compared to Ad5. Since liver is an interesting target organ for gene therapy, we then checked three hepatocellular carcinoma originated cell lines. While in HepaRG and HepG2 the three new viruses showed low transduction, Ad70 and Ad73 revealed around 40% transduction rates of Ad5 levels in Huh7 cells. In a further panel of breast cancer cell lines, we observed tumor type dependent transduction. All three viruses only poorly transduced the two triple-negative breast cancer cell lines Hs 578T and MDA-MB-231, while MCF7 cells showed 40% and 60% transduction rates compared to Ad5. As control, transduction of the three most commonly used adenovirus propagation cell lines HEK 293, A549, and Hela were included.

### 2.6. Screening of the Receptor Usage

**Figure 6:**
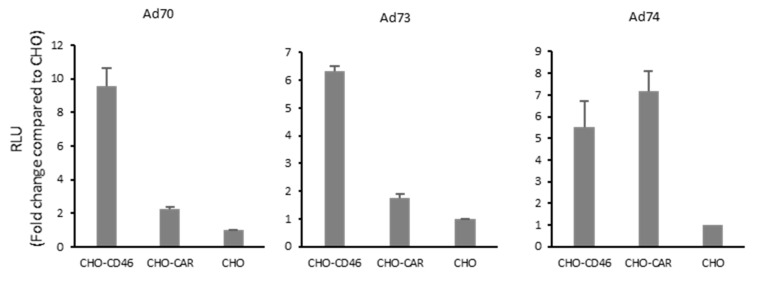
Receptor usage screening of new species D human adenoviruses. Cells were infected at 100 viral particles per cell and luciferase expression was measured 26 h post-infection by addition of furimazine substrate and expressed as relative light units (RLU). Levels were compared to the normal CHO cells and indicated as fold change. In all cell lines, error bars represent mean ± SD (*n* = 3).

To explore the major receptor usage of these new species D human adenoviruses, we applied them in CHO cells stably expressing cluster of differentiation 46 (CHO-CD46), the human coxsackie-virus and adenovirus receptor (CHO-CAR), and CHO control cells (Figure 6). Transduction efficiencies evaluated by luciferase reporter assays were standardized by comparison with the CHO control cells. Ad70 and Ad73 revealed 10- and six-fold higher transduction rates in CHO-CD46 cells compared to CHO control cells, respectively. This indicates that both viruses can use CD46 as an entry receptor. With respect to Ad74, we observed that it can transduce both CD46 and CAR positive cell lines with five- and seven-fold higher efficiency, indicating that Ad74 can use both CD46 and CAR as receptors.

### 2.7. New Species D Human Adenoviruses with Lower or Comparable Prevalence of Anti-Adenoviral Antibodies

**Figure 7:**
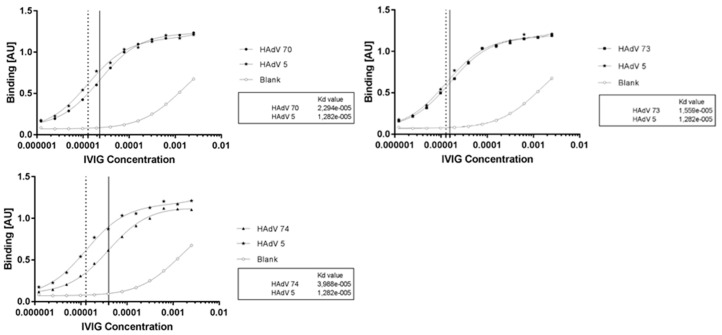
Analyses of antibodies directed against new species D human adenoviruses. ELISA experiment was performed to measure the binding affinities of adenovirus to intravenous immunoglobulin (IVIG) containing IgG antibodies from approximately 10.000 healthy donors. After curve fit evaluation via GrapPad Prism using One Site—Total method the dissociation constant (Kd) was drawn into the sigmoid curves.

Besides receptor usage, pre-existing immunity is another factor limiting broad application of some adenoviral vectors, such as Ad5. Here we measured binding affinities of Ad70, Ad73, and Ad74 to immunoglobulin G (IgG). Figure 7 shows the binding affinity curve of each virus compared to Ad5. We found that Ad70 and Ad73 have comparable biding affinity to Ad5, and the Ad74 was much lower than Ad5. Note, the binding affinity revealed level of total antibodies to individual adenovirus.

## 3. Discussion

Advanced genetic modification techniques to clone complete adenovirus genomes and to subsequently introduce modifications are essential to investigate emerging adenovirus pathogens. Furthermore, this is the first step for generation of customized vectors for gene therapy, tumor therapy based on oncolytic adenoviruses, and for genetic vaccination. That direct cloning of adenovirus genomes utilizing RecE/RecT recombination procedures in *E. coli* can lead to the generation of a cloned adenovirus library from species A to G was previously documented [22]. The process is well established when using genomes isolated from purified virus. A further step, to advance this cloning procedure, is utilizing a DNA mixture with complexities of various infectious materials, such as infected cells or patient samples.

Here we optimized adenovirus genome direct cloning by exploring the functional window regarding homologous arm (HA) length between 30- to 500-bp and genomic DNA input from 10^10^ down to 10^5^ copies. Previously, in the discovery of using full-length RecET for linear-linear homologous recombination (LL-HR), Fu and colleagues observed increased cloning efficiency in concert with extending the HA length (obtained colonies numbers from 415 for 20 bp HA to almost 200,000 with 120 bp HA). Using a linear cloning vector with the 50-bp HA length, they performed cloning of linear fragments from 10 to 51 kb and observed reduced efficiency as the fragments size grew [25]. It is of note, in those experimental settings both cloning components contained an antibiotic-resistant gene, which had positive influence in the cloning efficiency. In the current study, the first component in LL-HR is the antibiotic-resistant gene containing the cloning vector p15A-cm-Ad9HA, and the second is adenovirus genome. Considerable cloning efficiency and accuracy were obtained in the evaluated HA range of 30- to 500-bp. Similar to the double antibiotic-resistant gene recombination cloning, we also observed consistent increasing of cloning efficiency and accuracy as the HA length extended (Figure 1c,d). As the initial DNA input also plays an essential role in the cloning procedure, we then analyzed the effect of the adenovirus genome amount regarding cloning efficiency and accuracy. We found that the likelihood to obtain positive clones sharply dropped as the virus genome copy number (VCN) reduced from 10^10^ to 10^9^. With VCN lower than 10^9^ none of the settings gave positive results (Figure 2b,c). This finding is also important for testing cloning of virus genomes directly from clinical samples. Because the amount of adenovirus genomes in the samples are limited, to achieve successful cloning, it should at least exceed this limit of VCN.

Furthermore, the ability to directly clone from total DNA preparation of adenovirus infected cells was verified by using four newly identified species D adenoviruses (Ad70, Ad73, Ad74, and Ad75). Species D adenoviruses with 73 types is so far the largest sub-group of HAds. Previous studies revealed recombination between species D types and selection of novel neutralization epitopes (‘immune escape’) as major causes of this huge diversity in the adenovirus species [26,27]. Most of the species D adenoviruses were isolated from the stools of immunocompromised patients, such as AIDS patients or hematopoietic stem cell recipients [5]. The hypothesis is that the homologous recombination between two or among multi-types occurs in the gastrointestinal tract, where numerous diverse microorganisms live. There are hints that the bacterial Rec enzymes from *E. coli* enabled the recombination in immunocompromised individuals [28,29]. Multiple alignments of the current four species D types also revealed highly conserved sequences (Figure 3a and Figure 4a). Therefore, we were able to perform the genome cloning and further modification in a high-throughput way with shared vector cloning design. Again, here the quantified VCN played an important role in cloning accuracy (Figure 3b).

To exemplify the broad applications of adenovirus genome cloning and modification, we performed screening using the luciferase reporter. Transduction of various cancer cell lines derived from colon, liver, and breast cancer carcinoma origins demonstrated lower infection rates of these new adenoviruses than Ad5 (Figure 4). Since the tropism of adenovirus is largely determined by the capsid proteins that interact with the host cell surface molecules, individual adenoviruses frequently share the similar receptor usage within their species [30]. Currently, numerous molecules, such as the coxsackie and AdV receptor (CAR), CD46, desmoglein-2 (DSG2), sialic acid, integrins, CD80, CD86, vascular cell adhesion molecule-1 (VCAM-1), heparan sulfate proteoglycans (HSPGs), major histocompatibility complex class I-α2 (MHC-I-α2), dipalmitoylphosphatidylcholine, lactoferrin, and others have been confirmed to be utilized by adenoviruses to transduce cells [31]. Meanwhile, several adenovirus types were verified to use more than one major receptor for cell entry, such as Ad3 can use both DSG2 and CD46, Ad17 can use both CD46 and CAR, Ad37 can use both CD46 and sialic acid [32,33,34,35]. With respect to Ad52, its bi-receptor usage of CAR and sialic acid is due to the fact that it has of two types of fibers [36]. In the current study, screening of adenovirus receptors expressing CHO cells indicated that Ad70 and Ad73 use CD46 as cellular entry receptor, while Ad74 can use both CAR and CD46 for cell entry (Figure 5).

Previous studies uncovered a high prevalence anti-adenovirus antibodies to Ad5 and other common adenovirus types [15,16,37]. The preexisting immunity may preclude efficient gene transfer and the immune responses against the used vector for instance in gene therapeutic studies [38]. Therefore, it is important to analyze the existence of anti-adenovirus antibodies of any new isolate. Here the antibody binding test against pooled and concentrated human IgGs showed that at least one of these novel clinical isolates (Ad74) were less prevalent than the commonly used Ad5 vector (Figure 6).

In summary, direct cloning of adenovirus genome from infected cells can bypass large-scale virus amplification and purification. This will on one-hand access clinical samples in high accuracy, which can avoid multi-tissue culture procedures that may induce potential mutations in the adenovirus genome. On the other hand, it enables the discovery of new adenovirus types before they are isolated. For example, after detection of certain adenovirus species, a species-specific cloning vector can be applied to directly clone the related genomes before individual virus isolation. However, pre-culture in mammalian cells is still required in the current approach. In the future development, direct cloning from patient samples is of major interest.

## 4. Materials and Methods

### 4.1. Generation of Linear Cloning Vector p15A-cm-Ad9HA

A pre-generated plasmid p15Acm-Ad9ccdB was used as template [22]. To generate the cloning vector with different homology arm (HA)-lengths, primers binding to individual location (Figure S1, Table S1) were used. PCR was conducted with PrimeSTAR^®^ Max DNA Polymerase (Takara, Saint-Germain-en-Laye, France) according to manufacturer’s suggestion.

### 4.2. Genomic DNA Isolation for Adenoviral Genome Cloning

Cesium chloride (CsCl) gradient purified adenovirus or adenovirus infected A549 cells (two days after infection) were lysed for 2 h with proteinase K–SDS solution pH7.5–8 (TE buffer, 0.5% SDS, 500 µg/mL proteinase K) at 56 °C, with low speed shaking (300 rpm). Then the total DNA was extracted with phenol:chloroform:isoamyl alcohol (25:24:1. Carl Roth, Karlsruhe, Germany), followed by ethanol precipitation.

### 4.3. Linear-Linear Homologous Recombination (LL-HR)

LL-HR was performed in L-arabinose (Sigma-Aldrich, Steinem, Germany) induced *E. coli* strain GB05-dir as previous described [21]. Briefly, 500 ng of cloning vector and 500 ng of gAd or gAd mixed with eukaryotic genomic DNA were co-electroporated into fresh prepared competent *E. coli* GB05-dir, followed by antibiotic selection with chloramphenicol.

### 4.4. Colony-Polymerase Chain Reaction (PCR) Detection

Single clone was first picked for 1 h culture in chloramphenicol containing LB-medium. Then 1 µL was used as template for PCR with OneTaq DNA Polymerase (NEB, Berlin, Germany). Primers diag9-fwd and diag9-rev detect the gAd9, while CMR-fwd and CMR-rev amplify the cloning vector backbone (Figure S1, Table S2).

### 4.5. Quantitative Real-Time PCR (qPCR) to Detect Virus Genome Copy Numbers (VCN)

To quantify VCNs, qPCR with primers Ad-qPCR fwd/rev (Table S2) was performed with my-Budget 5× EvaGreen^®^ QPCR-Mix II reagent (Bio-Budget, Krefeld, Germany) according to the manufacturer’s protocol. The PCR cycles were performed and detected in the CFX Connect Real-Time PCR Detection System from Bio-Rad (Duesseldorf, Germany).

### 4.6. Cell Cultures

Human cell lines A549, Caco2, HCT116, HEK 293, Hela, HepaRG, Hs 578T, Huh7, MDA-MB-231, MCF7, SK-BR-3, T84 and Chinese hamster ovary cells CHO-CAR, CHO-CD46, and CHO control cells were cultured in high-glucose Dulbecco’s Modified Eagle’s Medium (DMEM, PAN-Biotech, Aidenbach, Germany). Huh7 cells and all three CHO cells were supplemented with non-essential amino acids (PAN-Biotech, Aidenbach, Germany). CHO-CAR and CHO-CD46 cells were selected with 50 µg/mL G418. HepG2 cells were cultured in RPMI 1640 (PAN-Biotech, Aidenbach, Germany). HepaRG cells were cultured in William’s E Medium (PAN-Biotech, Aidenbach, Germany). Breast cancer cell line SK-BR-3 was cultured in McCoy’s 5A Medium (PAN-Biotech, Aidenbach, Germany). All the above media were supplemented with 10% (except 20% for CaCo2) FBS, (GE Healthcare, Solingen, Germany), 100 units per mL (U/mL) penicillin, and 100 µg/mL streptomycin (PAN-Biotech, Aidenbach, Germany). All cells were maintained in a humidified atmosphere at 37 °C and 5% CO_2_.

### 4.7. New Species D Human Adenoviruses

New species D human adenovirus types 70, 73, 74, and 75 were clinical isolates. Detailed information is summarized in the Table 1 below.

### 4.8. Adenovirus Production

For the adenovirus reconstitution, amplification, purification, and titration, we followed standard protocols as previously described [39,40]. In brief, the adenoviral genome was released from the plasmid backbone with pre-inserted restriction enzymes, which do not cut the respective adenoviral genome (here, the PacI and PmeI are of choice). Then the adenovirus was reconstituted by transfection of a permissive cell line (HEK 293 cells). After serial passaging to amplify the adenovirus to large-scale, it was purified by CsCl (Sigma-Aldrich, Steinem, Germany) gradient in Beckman ultra-centrifuge (Krefeld, Germany). To determine the physical titer, viral DNA was released from virions obtained from CsCl gradients in dilution buffer (100 mM Tris, 10 mM EDTA, 0.1% SDS). The absorbance at 260 nm was measured on spectrophotometer (Eppendorf AG, Hamburg, Germany). The optical particle units (OPU) for adenoviruses were calculated using the following formula: OPU/mL = (absorbance at 260 nm) × (dilution factor) × (1.1 × 10^12^) × (36)/(size of adenovirus genome in kb).

### 4.9. Evaluation of Adenovirus Transduction Efficiency via Luciferase Assay

The transduction efficiencies of adenoviruses in different cell lines were measured by determining reporter gene (luciferase) expression levels. Individual tumor cells were grown to confluency in 96-well tissue culture plates and infected with different viral partial numbers (vp) per cell. Precisely, 24 h after infection, luciferase activity was measured with the Nano-Glo Assay System (Promega, Mannheim, Germany), and luminescence was detected with a plate reader (Tecan, Crailsheim, Germany).

### 4.10. ELISA to Measure Presence of Anti-Adenovirus Antibodies

For the ELISA 96-well plates were used. The experiment was performed with human IVIG (intravenous immunoglobulin, pooled and concentrated human IgGs. Privigen, Hattersheim am Main, Germany) diluted 1:300. The viruses were used at 3 × 10_6_ virus particles per well. As a positive control, wells were treated with coating buffer only. After incubation of the different samples with coating buffer overnight at 4 °C, the plates were washed two times. In the next step, wells were blocked with blocking buffer for 45 min at room temperature. After five times washing IVIG (pre-diluted, 1:300 in DPBS with 5% BSA) was added. IVIG was then successively diluted 1:2 in blocking buffer (DPBS with 5% BSA) 11 times. The plate was incubated at 37 °C for one hour. After washing, we added diluted second antibody (1:2000, Goat pAb Hum IgG (HRP), Abcam, Cambridge, UK) in blocking buffer to the wells and incubated the plate at 37 °C in a wet chamber for 70 min. Then the plate was washed five times again. After adding 100 µL substrate solution (SIGMAFAST™ OPD, St. Louis, MO, USA) to each well the plate was incubated in a dark chamber for 5 min. Finally, we added 100 µL H_2_SO_4_ per well to stop the reaction. The plate was measured with Tecan ELISA Reader at a wavelength of 492 nm.

The evaluation of ELISA results was carried out with the statistic software GrapPad Prism (Version 7.04, GraphPad Software, San Diego, CA, USA). After curve fit evaluation the dissociation constant (Kd) was determined according to the Michaelis–Menten model and drawn into the sigmoid curves.

### 4.11. Statistics

Statistical analyses were conducted with Microsoft Excel. Experimental differences were evaluated by Student’s *t*-test.

## Figures and Tables

**Table 1:** Summary of New Species D Human Adenoviruses used in this study.

Virus	NCBI Access Number	Penton/Hexon/Fiber	Date/Place	Source (Isolated From)
Ad70	KP641339	P70H70F29	2014/Leipzig, Germany	Diarrheal feces of a hematopoietic stem cell transplantation recipient [23]
Ad73	KY618676	P67H45F27	2015/Leipzig, Germany	Diarrheal feces of a lymphoma patient treated with chemotherapy [24]
Ad74	KY618677	P70H74F51	2015/Leipzig, Germany	Diarrheal feces of a hematopoietic stem cell transplantation recipient [24]
Ad75	KY618678	P75H26F29	2015/Leipzig, Germany	The feces of an AIDS patient [24]

## Supplementary Materials

Supplementary Materials can be found at https://www.mdpi.com/1422-0067/21/17/6370/s1. Table S1. Oligonucleotides for amplification of p15A-cm linear vectors. Table S2. Oligonucleotides for detective PCR. Figure S1. Generation of cloning vector with increased HA-length: ~30, ~50, ~100, ~150, ~200, ~500 bp. Figure S2. Positive clone detection.

## Abbreviations

CAR	Coxsackievirus and adenovirus receptor
CD46	Cluster of differentiation 46
CsCl	Cesium chloride
CV	Cell/virus lysate
E3	Early gene 3
gAd	Human adenovirus genome
GFP	Green fluorescent protein
GLN	Turbo GFP, nanoLuciferase (Nluc), and neomycin resistance (neo) reporter cassette
HA	Homology arm
HAdV	Human adenovirus
IgG	Immunoglobulin G
IVIG	Intravenous immunoglobulin
LLHR	Linear-linear homologous recombination
ITR	Inverted terminal repeat
MLP	Major late prompter
PCR	Polymerase chain reaction
RLU	Relative light units
VCN	Virus genome copy number
