# Supplementary material for: High-Throughput Cloning and Characterization of Emerging Adenovirus Types 70, 73, 74, and 75
Source: Int J Mol Sci. 2020 Sep 2;21(17):6370. doi: 10.3390/ijms21176370 (PMC7504450; doi:10.3390/ijms21176370)
Supplement: Supplementary file 1 [file ijms-21-06370-s001.pdf]

Table S1. Oligonucleotides for amplification of p15A-cm linear vectors

Name	Sequence	Note /application
Ad9-HA30-F/R	ttactttgtggggtatattatatagatag	p15A-cm-Ad9HA30 PCR
Ad9-HA50-F/R	gctcatttgcatattaacttttg	p15A-cm-Ad9HA50 PCR
Ad9-HA100-F/R	tcgtccaatcagcgttggct	p15A-cm-Ad9H100 PCR
Ad9-HA150-F/R	cgaccgttagccgtgcgt	p15A-cm-Ad9HA150 PCR
Ad9-HA200-fwd	cgccatgttgcccacgtcca	p15A-cm-Ad9HA200 PCR
Ad9-HA200-rev	gcgacttgcttccgggctag	
Ad9-HA500-fwd	aagcatacagagattcagccata	p15A-cm-Ad9HA500 PCR
Ad9-HA500-rev	acggactcgactggtttaaatac	
Ad-D70-HA-fwd	Agctcatttgcatattaacttttgtttactttgtggggtatattattgatgatg CCTGCAGGATTTAAATTAATTAAGCGA	p15A-cm-Ad70HA50 PCR
Ad-D70-HA-rev	Agctcatttgcatattaacttttgtttactttgtggggtatattattgatgatg GTTTAAACGGCCGGCCTAG	

Table S2. Oligonucleotides for detective PCR

Name	Sequence	Note /application
diag9-fwd	atcttcctggggcatctctt	Diagnostic PCR to detect E2A DBP of Ad9
diag9-rev	tgtttcagcagcacatcctc	
CMR-fwd	TGGAGTGAATACCACGACGA	Diagnostic PCR to detect plasmid backbone
CMR-rev	ATCACAGACGGCATGATGAA	
Ad-qPCR fwd	caggacgcctcggagtacctga	Viral genome copy number quantification
Ad-qPCR rev	gggccaccgtggggttc	

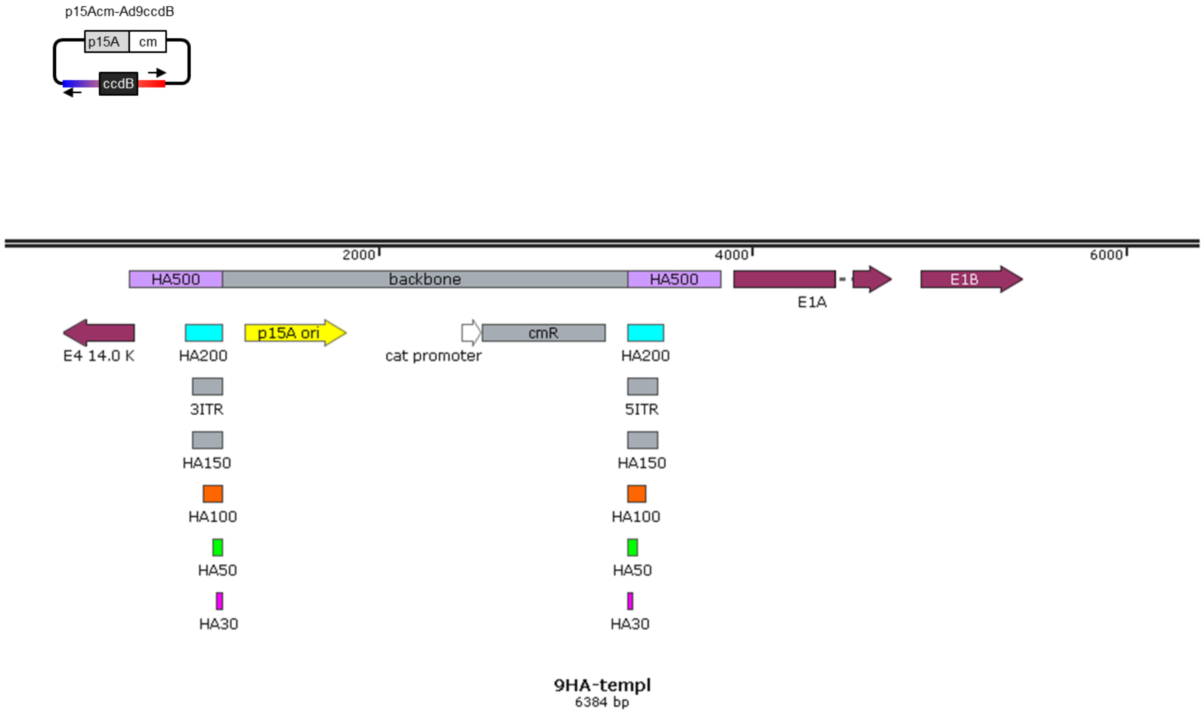

Figure S1. Generation of the cloning vector with increased HA-lengths: ~30, ~50, ~100, ~150, ~200, ~500 bp. Scheme shows the position of homologous arms. A pre-generated plasmid p15Acm-Ad9ccdB was used as template. This plasmid is gAd9 cloned into p15A-cm backbone, with a ccdB insertion in the E3 genome region to avoid plasmid contamination in PCR.

A

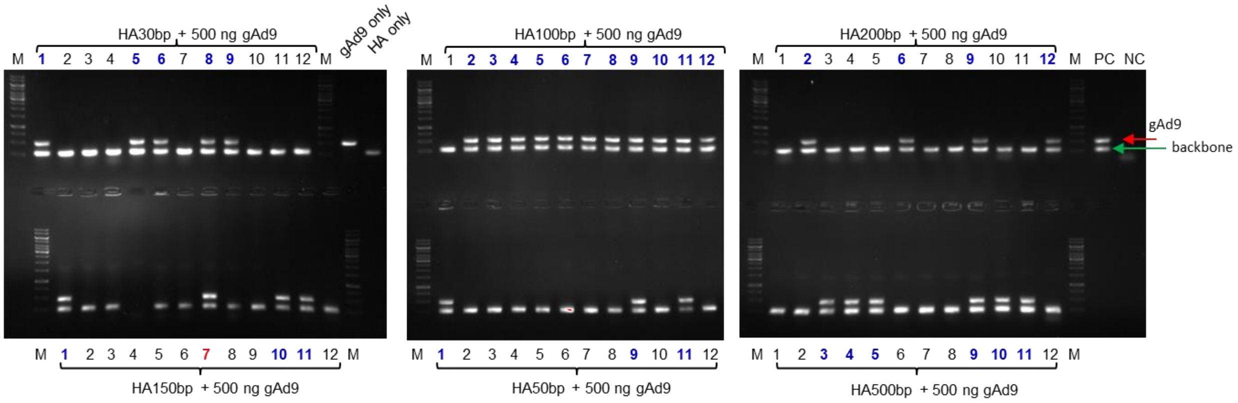

B

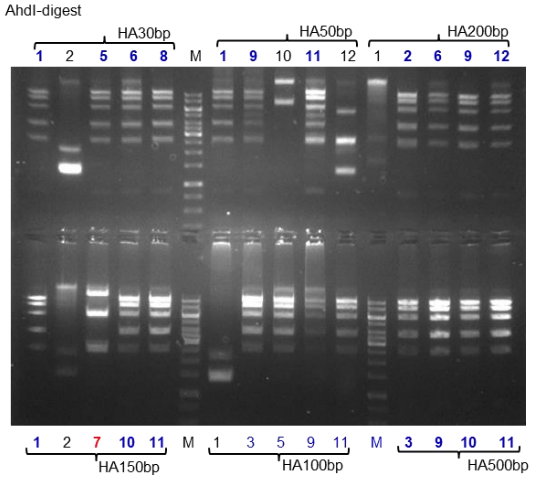

7/31/2020

C

Length (bp)	HA30		HA50		HA80		HA150		HA200		HA500	
gAd9 (ng)	500	50	500	50	500	50	500	50	500	50	500	50
Accuracy (Positive /Colony checked)	5/12	3/15	3/12	2/14	11/12	9/15	4/12	7/15	4/12	8/15	6/12	8/15
	2/12	0/15	5/12	2/15	8/14	6/14	7/14	6/15	7/13	5/15	11/15	8/15
		0/15		0/15	9/13	8/15	6/15	8/15	10/15			
					7/15							

Figure S2. Positive clone detection. **A.** Representative gel of colony-PCR to detect cloned gAd9. Presented are the analyses of 12 colonies per setting and a blue number indicates the clones to be gAd positive. gAd9, HA-only and a previously cloned Ad9 are included as control. The detection of gAd9 gives a 500-bp band, and the backbone detection gives a band of 250 bp in size. **B**. AhdI restriction analysis of the cloned adenoviral genome in p15A. Correct clones are indicated with a blue number, while the red number 7 in the HA150 group was a false-positive clone detected by colony-PCR. **C.** Summary of cloning accuracy.
